# Fields of inequity: initial insights into structural disparities among migrant farmworkers in Maryland

**DOI:** 10.3389/fpubh.2026.1930068

**Published:** 2026-10-01

**Authors:** Devon C. Payne-Sturges, Raul Cruz-Cano, Stephanie Hernandez, Zoe Brokering, Ellis Ballard, Thurka Sangaramoorthy, Leila Borrero Krouse, Jessica Culley

**Affiliations:** 1 Department of Environmental Health Sciences, School of Public Health, University of Michigan, Ann Arbor, MI, United States; 2 Department of Global, Environmental, and Occupational Health, University of Maryland School of Public Health, College Park, MD, United States; 3 Department of Epidemiology & Biostatistics, School of Public Health, Indiana University, Bloomington, IN, United States; 4 Department of Epidemiology, School of Public Health, University of Michigan, Ann Arbor, MI, United States; 5 Tributary Design, St. Louis, MO, United States; 6 Brown School of Social Work at Washington University in St. Louis, St. Louis, MO, United States; 7 Department of Anthropology, American University, Washington, DC, United States; 8 CATA – The Farmworker Support Committee, Glassboro, NJ, United States

**Keywords:** disparities, farmworkers, immigration, migrant housing, migrant labor, policy, respiratory health, structural inequities

## Abstract

**Background:**

Despite well documented health vulnerabilities of migrant and seasonal farmworkers (MSFWs), few public health studies have comprehensively evaluated these population health differences through the lens of social causal structures. In this article we describe a community-based participatory and interdisciplinary research partnership, RESPIRAR, a community-based and interdisciplinary research collaboration that seeks to understand the social, structural and institutional drivers of respiratory health and housing conditions of migrant and seasonal farmworkers (MSFWs) in the Mid-Atlantic States and we report on structured disparities among MSFWs in Maryland participating in our study. Few data exist on the demographics, health and housing conditions of MSFs currently employed in Maryland.

**Methods:**

All elements of RESPIRAR were co-designed by the partnership including the development of the baseline survey. Participant recruitment and data collection were carried out by teams consisting of staff from the university collaborators and CATA, a farmworker organization with over 40 years of experience supporting farmworkers and low-wage immigrant workers in the Mid-Atlantic region. 135 MSFWs on Maryland’s Eastern Shore completed baseline surveys on their demographic characteristics, housing and working conditions, and health status during the 2023 and 2024 agricultural seasons. In addition to generating summary statistics, we examined differences in wages and housing type by farmworker demographics, work authorization status and work tasks.

**Results:**

MSFWs held a variety of work authorization statuses (77.8% H-2A and 15% EAD under TPS), reported Spanish (84.3%) and Haitian Creole (15.7%) as primary languages, and 47% reported long tenure in agricultural work. Participants lived in various overcrowded housing types including detached/single family homes, motels, barrack/labor camps and mobile homes. Hypertension was the most common chronic health condition reported by participants but not everyone had access to needed medications. We found disparities in wages by gender, education, and nationality (e.g., male, H-2A and Mexican workers were significantly more likely to earn ≥$15/h.).

**Conclusion:**

Given the lack of systematically collected data on Maryland’s present-day MSFWs, our data provide critical insights for local farmworker advocacy, social service and health care organizations, and governmental agencies responsible for the health and wellbeing of MSFWs. Impoverished working and living conditions of MSFWs are constant features, structured by design of the United States agriculture labor system. To find lasting solutions, we can no longer treat these features as independent factors. We must apply a systems lens to examine how the structures of law, policy, practices and norms give rise to the lived experiences of MSFWs. Our baseline results provide grounding into the current lived experiences of MSFWs in Maryland and aspects of labor, immigration, agriculture and public health systems that we will interrogate further through community-based system dynamics and group model building workshops co-facilitated with CATA.

## Introduction

Although Maryland is not often thought of as a farming state, agriculture is Maryland’s second largest industry, generating about $8 billion in total economic activity for the state (1, 2) and a significant amount of farmland lies on Maryland’s Eastern Shore. Maryland’s agricultural sector has always relied on labor that was controlled, surveilled, exploited and subject to harsh working and living conditions. Workers who have contributed to Maryland’s agricultural economy include those who were enslaved, Black tenant farmers, German prisoners of WWII and present day migrant and seasonal farmworkers (MSFWs) (3). MSFWs come to Maryland mainly from Latin America (Mexico) and the Caribbean (Haiti and Jamaica) either as part of the Eastern Migratory Stream and/or under the H-2A guest work visa program. Maryland’s Department of Labor reports that fewer than 1,000 MSFWs are employed in the State’s agricultural fields during the growing season (4). This may be an underestimate. According to the U.S. Department of Labor 1,484 H-2A positions were certified for Maryland employers in 2025 and approximately 80% of H-2A certifications result in visa approvals. However these statistics do not include non-H-2A MSFWs working on Maryland farms. Regardless, mirroring national trends, the demand for H-2A workers has increased dramatically in Maryland, almost doubling from 777 H-2A certified positions in 2017 to 1,484 positions in 2025 (5, 6). Other studies have estimated up to 7,000 MSFWs entering Maryland each year to harvest fruit and vegetable crops, work in orchards, canneries and on nursery farms (7). With many MSFWs coming from out of state, a major problem in Maryland has always been the living conditions for these workers, who are often housed at labor camps and other housing locations owned or leased by farm owners and labor contractors.

The living and workplace conditions of MSFWs received renewed attention during the 2020 global pandemic, including in Maryland (8–13). Infectious respiratory disease outbreaks present challenges for the health and wellbeing of MSFWs: these workers are deemed “essential” due to their central role in supporting the local, national and global economies, farm owners and the food supply (14–17), yet MSFWs are among the lowest paid, most vulnerable workers, laboring and living under conditions that pose risks to their health ranging from crowded housing to a lack of PPE, field sanitation, and handwashing stations on work sites (18–24). Additionally, MSFWs lack access to health information, preventive care and medical treatment (20, 25–29). These vulnerabilities are often compounded by linguistic and legal barriers (25, 30). Viruses may spread quickly between MSFWs and to surrounding communities as migrant farmworkers move from farm to farm (31, 32). Indeed, outbreaks of respiratory diseases have been reported among farmworkers and agricultural counties in the United States at disproportionately high rates (9, 30, 33–35).

Airborne transmission plays a significant role in the spread of respiratory viruses, particularly in crowded indoor spaces with poor ventilation (36–38). Preventive measures recommended to the general public to reduce risk are structurally constrained by the segregated, substandard, and overcrowded housing conditions in which MSFWs live, and their often unsafe or limited water supply at worksites and housing locations, making hand hygiene and social distancing challenging, if not impossible (3, 22, 39–43). The undocumented status of many MSFWs and restrictions of H-2A visas render these workers especially vulnerable to abuse and exploitation and unable to effectively assert health-related rights, given the risk of retaliatory firing and potential deportation (29, 44, 45). These very same vulnerabilities were previously identified as barriers for successfully preparing and protecting farmworkers in times of a health crisis such as pandemic influenza, but were never comprehensively addressed (46–50). Despite these known health vulnerabilities of MSFWs, few studies have comprehensively evaluated these population health differences through the lens of social causal structures (SCSs) which we define as the macro level systems, social forces, institutions, laws, policies, practices, norms and processes that interact with one another to generate and reinforce adverse health outcomes within population groups (51–54).

This paper has two objectives. First, we describe the design for an interdisciplinary and community-based participatory research project examining the SCSs impacting MSFW respiratory health. Second, we describe baseline characteristics and examine differences in wages and housing type by demographics, work authorization status and work tasks among farmworkers in Maryland participating in our study. These results help to identify SCSs for more in-depth investigation through our planned legal analysis and system dynamics group model building workshops. Additionally, given the lack of systematically collected data on Maryland’s present-day MSFWs, reporting on our baseline data provides timely and critical insights for local farmworker advocacy, social service and health care organizations (including our community partner CATA), and governmental agencies responsible for the health and wellbeing of MSFWs.

## Methods

### Study design

The RESPIRAR Project, is an on-going, innovative, multilevel, interdisciplinary and community-based participatory research effort designed to illuminate the social, structural and institutional drivers of the respiratory health of MSFWs in the Mid-Atlantic Region of the United States. Our work combines environmental health science, community-based system dynamics (CBSD) (55), ethnographic and archival methodologies, statistical analysis and public health approaches with legal analysis to unpack the mechanisms through which SCS shapes respiratory disease trends among MSFWs. RESPIRAR has three aims: (1) examine the relationships between migrant labor housing, indoor air quality, exposure to respiratory viruses and respiratory health of Maryland MSFWs over time; (2) characterize pathways through which various policy and regulatory responses interact and influence health outcomes among MSFWs in Mid-Atlantic states using a community-based and participatory system dynamics group modeling approach; and (3) evaluate the influence of labor and occupational health and safety laws and policies on infectious respiratory disease outcomes across selected agricultural states using legal epidemiology and a quasi-experimental study design.

Our research is a committed partnership between university-based researchers and the community-based organization Comite de Apoyo a los Trabajadores Agricolas (CATA), a farmworker organization with over 40 years of experience supporting farmworkers and low-wage immigrant workers in the Mid-Atlantic region. Additional collaborators include students, scientists and scholars with expertise in exposure assessment, public health, labor and employment law, systems science, community-based participatory research, policy analysis, anthropology, environmental microbiology, virology, epidemiology, bioinformatics and statistics. We established a Community Advisory Committee (CAC) to serve as advisors in the interpretation and communication of research findings and to contribute to the research effort by participating in CBSD group model building workshops. The CAC includes representatives from three leading farmworker advocacy organizations [Farmworker Justice, Migrant Clinicians Network (MCN), and Centro de los Derechos del Migrante, Inc. (CDM)], and representatives from local hospital systems, a local farm owner and a local health department. The RESPIRAR team maintains regular engagement with CAC through research update meetings (including preliminary review of findings presented in this article) and electronic communications in addition to hosting a training workshop in 2025 on CBSD methodology with members of the CAC. The ultimate goal of RESPIRAR is to identify high-leverage target areas to focus change efforts and specific strategies for mitigating infectious disease risk among agricultural workers at their worksites and in employer provided housing. To this end, we have continuously leveraged opportunities to share research results and evidence-based recommendations to state law makers and state health, labor and environment agencies responsible for the health and safety of MSFWs. For example RESPIRAR recently provided an educational briefing on migrant farmworker housing conditions to the House Economic Matters Committee (EMC) of the Maryland General Assembly upon invitation by the EMC Chair.

One component of the RESPIRAR Project is an intensive field study on Maryland’s Eastern Shore conducted during the 2023 and 2024 agricultural seasons to examine the relationships between migrant labor housing, indoor air quality, exposure to respiratory viruses and respiratory health of MSFWs over time using repeated symptom surveys and laboratory tests of weekly nasal swabs. The longitudinal study also included the collection of baseline information on demographics, housing and working conditions, and health status through questionnaires completed by farmworker participants. This article focuses on the baseline data collected as part of the longitudinal study. The study protocol was approved by the Institutional Review Board at the University of Maryland.

### Participant recruitment

Farmworker participants were recruited and enrolled during the 2023 and 2024 growing seasons (March through October) on Maryland’s Eastern Shore in the Mid-Atlantic region of the United States, which consists of nine counties covering a total land area of approximately 3,674 square miles (56): Caroline, Cecil, Dorchester, Kent, Queen Anne’s, Somerset, Talbot, Wicomico, and Worcester (Supplementary Figure S1). The North-to-South length of Maryland’s Eastern Shore is approximately 110 miles. Since there is no current official count of MSFWs in Maryland, we used MSFW housing locations as our sample frame. We identified 36 housing locations from publicly available information on H-2A certifications from the U.S. Department of Labor and locations known to the Maryland office of CATA, our community-based partner. Twenty-two locations refused to participate, could not be verified/located, were unsafe to visit, did not house migrant workers or housed workers who were already leaving and thus not able to participate. This resulted in 14 unique housing locations agreeing to participate – a 39% housing location response rate. Thus we recruited a convenience sample of farmworkers at farm labor camps and housing locations on a rolling basis throughout the growing season. Recruitment was carried out by research teams from our universities and CATA. In order to enroll in our study, participants had to be at least 18 years of age, currently employed in agricultural work, migrating for employment, and residing in migrant labor housing or at a migrant labor camp.

### Data collection

All project partners contributed to survey questions and protocol, guided by the principles of community-based participatory research. Survey questions were developed to capture baseline demographic information, living conditions, transportation, work and wage characteristics, pesticide safety and hygiene practices, health behaviors, health status and respiratory symptoms (recent past and current). Items from previously published studies on farmworkers (6, 57–64) and infectious respiratory pandemics (60) were used whenever possible. Working together with CATA to develop the survey ensured results would also be useful for their programmatic needs. The baseline and weekly symptom (longitudinal study) questionnaires were developed in English and translated into Spanish and Haitian Creole. All three versions of the questionnaires were reviewed by native speakers from the University of Maryland and CATA field study team to ensure the meanings of words were correct and adjustments were made if needed. Study data were collected and managed using REDCap, a secure, web-based software platform designed to support data capture for research studies (65, 66).

Study recruitment and data collection teams included native Spanish and Haitian Creole speakers from University of Maryland and CATA who received CITI ethics training and training on how to consent participants and properly administer the questionnaires using REDCap. Additionally, team members were instructed on how to take field notes to collect qualitative information on their observations and conversations in the field following established ethnography methodologies (67, 68). Participant consent was obtained in writing. Once consented, participants were assigned a unique ID and completed the baseline questionnaire independently on individual iPads with REDCap installed in either Spanish or Haitian Creole and away from group settings, allowing for privacy and minimizing the potential of group influence on self-reported responses. If needed, trained study staff with Spanish or Haitian Creole language skills would read the consent form and questionnaire to the participant. All responses to questionnaires were self-reported. The baseline questionnaire took on average 45 min to complete, and participants were given a monetary incentive to compensate for their time and participation. REDCap questionnaires were completed at participants’ housing locations after work hours. Employers were not present when participants were enrolled and administered the baseline questionnaire.

### Measures

For the purposes of this paper, we present the analysis of eight measurement categories from our baseline questionnaire: (1) participant demographics; (2) work and wage characteristics; (3) crops and job tasks; (4) housing characteristics; (5) transportation; (6) pesticide exposures and hygiene practices; (7) heath behaviors; and (8) health status.

Demographic characteristics included gender (male, female), age in years, marital status (single, married, living with partner, widowed, divorced or separated), national origin (United States, Mexico, Haiti, other), educational attainment (less than primary school, primary school, secondary school, preparatory school), last locale before coming to Maryland (Delaware, Florida, Georgia, Mexico, New Jersey, Texas, Virginia) and preferred language (English, Spanish, Haitian Creole, Other).

Work characteristics included years worked in agriculture (less than 2 years, 2–3 years, 4–7 years, 8 or more years) and whether participants had worked as hired farmworker for at least 1 month in the United States during the previous year (yes/no). Additional categorical variables included number of weeks worked in the last 3 months (1–2, 3–4, 5–7, 8–11, 12 weeks), usual number of days worked per week (1–2, 3–5, 6–7 days), and hours worked in the last week (3–20, 21–31, 32–40, 41–69 h). Participant wage characteristics were captured using the following: method of payment (cash, check, other/direct deposit), whether they were paid by the piece or hour, and if paid by hour, their hourly rate ($7.25–$7.99, $8.00–$8.99, $9.00–$9.99, $10.00 - $14.99, $15.00 - $15.99 or $16.00 or greater). Among those paid at piece rate, we asked whether their actual work hours were recorded, and their equivalent hourly rate. Measures of “work downtime” (time at work during which they could not work) and whether they were paid for that time included time spent traveling from field to field, waiting for crops to dry, waiting for equipment repair, when sick or injured, when it was too hot/extreme heat, when there was a thunderstorm or lightning, and during an air quality advisory.

The North American Industrial Classification System (NAICS) defines farmwork as consisting of codes 111 (Crop Production), 112 (Animal Production), and 115 (Support Activities for Agriculture and Forestry) (56). Crop production includes not only outdoor farming, but also working in nurseries producing crops, seedlings, flowers, trees/seedlings, shrubs, flowers and grasses. Support activities include tasks such as sorting, grading, cleaning, packing, and packaging fruits and vegetables. For the purpose of this study, our definition of an agricultural worker excludes code 112 (Animal Production). We nonetheless use the term “farmworker” interchangeably with “agricultural worker,” which informed the categorical measures on crops and job tasks we used. Participants were asked to report the crops they currently worked in: apples, tobacco, berries, tomatoes, sweet potatoes, green peppers, squash, hot peppers, cucumbers, melons (incl. watermelons), nursery plantings (incl. Trees/seedlings, shrubs, flowers, grass) and other (which participants were asked to write in). Participants could select more than one category of crop. Free-text responses provided under the “other” crop category were reviewed and recoded when they corresponded to existing predefined categories (e.g., watermelon/sandía reassigned to melons). Responses reflecting non-crop activities (e.g., plant maintenance, grass, or tree removal) were excluded. This ensured mutually exclusive crop categories and improved interpretability of the final crop distributions.

Categorical measures for tasks the participants were performing currently included harvesting/picking, pulling weeds, loading, planting, driving vehicle (other than tractor), setting up stakes, driving tractor, irrigating, applying pesticides, pruning, and other tasks (which participants were asked to write in). Participants could select multiple tasks.

Baseline housing characteristics assessed included type of housing (single-family house, motel, mobile home/trailer, barracks/labor camp), whether the housing was owned by employer (yes/no), whether the farmworker paid for the housing (yes/no) and if so, what was the rent per month ($), number of bedrooms, number of bathrooms, number of kitchen/cooking areas, number of adults living in the housing, whether employer provided meals (yes/no), and whether certain amenities were present (yes/no) in the housing (hot/cold water and indoor plumbing, toilets, shower/tub, stove, refrigerator, central heating, central air, AC window unit, fans, laundry facilities, telephone/landline and WIFI/internet) and if they were functional (yes/no).

Migrant and seasonal farmworkers are often housed in very isolated locations (69–71) and must rely on their employers or crew leaders for transportation, not only for work but also for activities of daily living such as shopping for food and accessing health care. We therefore collected data on usual mode of transportation for work, shopping, and accessing health care (ride/"raite”; own vehicle; friend/relative; boss, contractor or farmer; other rideshare; bus or van of the ranch/farm; or public bus), if workers paid for transportation (yes/no) and how much ($), and length of commute (minutes).

Important occupational hazards that farmworkers face include pesticide exposures, lack of access to field sanitation and poor pesticide safety behaviors. Not only does poor quality housing increase the risk for the transmission of infectious disease, but also exposure to toxicants such as pesticides (31, 42, 63, 72–75). Pesticide exposure measures, in the context of participants’ current job, assessed whether they were currently working in areas that had been treated with pesticides (yes/no), have loaded, mixed or applied pesticides (yes/no), cleaned or repaired containers or equipment used for applying or storing pesticides (yes/no), breathed pesticides in the air (yes/no), touched plants with pesticide residue (yes/no), and been sprayed with pesticides by a plane or tractor (yes/no).

The EPA’s Worker Protection Standard regulates the use of pesticides in agriculture and has multiple requirements including: pesticide safety training, notification of pesticide applications, use of personal protective equipment, restricted entry intervals following pesticide application, decontamination supplies, and emergency medical assistance to reduce the risk of pesticide poisonings and injuries among agricultural workers and pesticide handlers (76). Typically state departments of agriculture are responsible for implementing the Worker Protection Standard. Given these regulations, our study also included questions on whether participants had received health and pesticide safety training at their current job and how that training was delivered (with an instructor/trainer in person, by watching a video or was a booklet given to read). According to the Worker Protection Standard, the minimum time delay for re-entering fields treated with pesticides is 4 hours after the pesticide application is completed, unless the pesticide label specifies a longer restricted-entry interval (REI). Since we did not collect pesticide specific information, we asked participants whether they have entered the field within 4 h after pesticide treatment as a measure of minimum safety standard. Availability of field sanitation supplies (handwashing, drinking water, disposable cups, bathrooms within 1/4 mile walking distance) were also assessed. Lastly pesticide safety behaviors assessed included whether participants remove boots before entering their housing (yes/no), the number of minutes after entering their housing from worksite they change clothing and bathe, and whether they wash their work clothes separate from other clothes (yes/no).

Health behaviors assessed included measures of tobacco use (yes/no ever smoked cigarettes, number of years smokes, number cigarettes smoked per day), alcohol consumption (how often drink per week, number of drinks per day), sleep (hours sleep during the week and during the weekend), and prescription medication use (yes/no).

Chronic health conditions such as high blood pressure (hypertension) and diabetes increase the risks of lower respiratory infections including pneumonia, COVID-19 and influenza, and have been associated with more severe complications and hospitalizations (77–80). Therefore we collected data on whether workers had been diagnosed (yes/no) with chronic illnesses such as chronic bronchitis, asthma, diabetes, kidney disease, hypertension, coronary heart disease, and chronic liver disease. We also asked participants to report respiratory systems within the last year (yes/no) such as wheezing or whistling in their chest, chest tightness, shortness of breath not associated with exertion, cough at night, and if they currently experience cough and phlegm first thing in the morning.

### Analysis

Descriptive statistics were generated for all baseline demographic, housing, work, wages and health-related variables. For continuous variables, means and standard deviations were summarized, and two-sample *t*-tests were used to compare values by demographic characteristics. For categorical variables, frequency distributions and percentages were calculated, and Fisher’s exact tests were used to assess demographic differences to accommodate small cell counts in some cases. Specifically we examined differences in wages (<$15 vs. ≥ $15/h.) by demographics, employment authorization, work characteristics and tasks. We dichotomized wages at $15/h. (Maryland’s minimum wage) to address sparsity across multiple wage categories and to facilitate stable estimation of regression models. We also examined differences in housing type by participant demographics, work authorization and work tasks. All analyses were conducted in Stata (version 19.0), and two-sided *p*-values < 0.05 were considered statistically significant.

## Results

### Demographic characteristics

One hundred thirty-five farmworkers enrolled in our study across 14 unique farmworker housing locations in seven of the nine Maryland Eastern Shore Counties. Fifty-four percent of participants completed surveys during early season (March–June), 41% during mid-season (July–August) and 5% during late season (September–November). As shown in Table 1, the majority of study participants were male (90%), married or had a partner and family they were supporting elsewhere (82%), completed secondary education or higher (74%), and were 44 years of age on average. While most participants were from Mexico (81.5%), a substantial proportion were from Haiti (15.6%). Only 2.2% were born in the United States. Mirroring the statistics for country of origin, the majority of participants were employed through the H-2A visa program (77.8%), while nearly 15% had work authorization as Temporary Protected Status (TPS) beneficiaries. Ninety-five percent of H-2A visa holders were from Mexico, 95% of TPS beneficiaries were for Haiti, and 5% of United States permanent resident workers were born in Mexico. Prior to arriving in Maryland for work, 73% were living in Mexico, 15.8% were living in Florida and 11.3% were living in either Haiti, Texas or South Carolina. Spanish was reported as the primary language for 84.3% of participants and Haitian Creole was reported for 15.7%.

**Table 1:** Migrant season farmworker demographic characteristics, Eastern Shore Maryland (*n* = 135).

Participant characteristics	*n*	% or Mean (SD)
Gender		
Male	122	90.4%
Female	13	9.6%
Age (Mean and SD years)		44 (13)
National origin		
Mexico	110	81.5%
Haiti	21	15.6%
United States (incl Puerto Rico)	3	2.2%
Other*	1	0.7%
Marital status		
Single	17	12.6%
Married	91	67.4%
Living with partner	19	14.1%
Widowed	4	3%
Divorced	1	0.7%
Separated	3	2.2%
Primary language		
Spanish	113	84.3%
Haitian Creole	21	15.7%
English	0	0%
Other/Indigenous	0	0%
Education		
Less than primary school (grades 0–5)	8	5.9%
Primary school (grade 6)	25	18.5%
Secondary school (grade 9)	66	48.8%
Preparatory school (grade 12) or greater	34	25.2%
Declined	2	1.5%
Last locale**		
Florida	21	15.8%
Mexico	97	73.0%
Other***	15	11.3%
Work authorization status		
H-2A Visa	105	77.8%
TPS EAD	20	14.8%
U.S. citizen	4	3.0%
Permanent resident	6	4.4%

* Peru; ** Before coming to Maryland, where were you living last?; *** Other included South Carolina, Texas, and Haiti.

### Work and wage characteristics

Nearly half of our study participants were veteran migrant and seasonal farmworkers (Table 2), returning to Maryland for seasonal farmwork for more than 8 years. Many reported consistently working for the same employer. At the time of baseline data collection, 14% reported having worked 1–2 weeks, 16% worked 3–4 weeks, 18% worked 5–7 weeks, 15% worked 8–11 week, and 33% worked 12 weeks or more. Participants generally reported working 6–7 days per week. However, hours worked varied with 13% reporting 3–20 h and 48% reporting working 41–69 h per week at the time of baseline survey. Among those reporting working 3–20 h per week (18 participants), 67% completed baseline surveys during early months of the growing season and 33% completed baseline surveys during the mid-season. In comparison, of the 65 participants reporting 40 h. per week or more, 45% completed baseline surveys during the early season and the mid-season.

**Table 2:** Work and wage characteristics (*n* = 135).

Work and wage characteristics	*n*	% or Mean (SD)
Number of years doing seasonal farmwork		
Less than 2 years	15	11.0%
2–3 years	27	20.0%
4–7 years	25	19.0%
8 or more years	64	47.0%
Decline	1	0.7%
Worked in Ag at least 1 month in the US during the last year		
Yes	111	82.0%
Number of weeks doing farmwork in the last 3 months		
1–2 weeks	19	14.0%
3–4 weeks	21	16.0%
5–7 weeks	24	18.0%
8–11 weeks	20	15.0%
12 weeks	45	33.0%
Decline	2	1.5%
Usual number of days worked per week		
1–2 days	3	2.2%
3–5 days	33	24.2%
6–7 days	99	73.4%
Decline	0	0%
Number of hours worked in the last week		
3–20 h	18	13.3%
21–31 h	8	6.0%
32–40 h	40	30.0%
41–69 h	65	48.0%
Decline	4	3.0%
Methods of payment		
Paid in cash	28	21.0%
Paid by check	96	71.0%
Other (direct deposit)	11	8.2%
By the hour or piece		
By the hour (Y)	133	98.5%
By the piece (Y)	1	0.7%
Decline	1	0.7%
Rate by the hour		
$7.25 - $7.99	2	1.5%
$8.00–$8.99	0	0%
$9.00–$9.99	0	0%
$10.00–$14.99	16	12.0%
$15.00–15.99	18	13.5%
$16.00 or greater	89	67.0%
Decline	8	6.0%
Hours recorded		
Actual work hours recorded (Y)	129	95.6%

Nearly all farmworkers in our study were paid by the hour. Slightly more than two-thirds (67%) of participants were paid $16/h. or more, a quarter were paid between $10 and $15.99/h., and 1.5% reported being paid well below Maryland’s $15/h. minimum wage at $7.25 - $7.99/h. Seventy-one percent were paid by check and 95% reported that their actual hours worked were recorded. Some participants reported instances out of their control when they were not able to work and were not paid. For example, 21.6% reported down time because they were sick or had an injury and of those, 62% said they were not paid during this time. Other instances included times when it was too hot, too much rain, bad air quality, traveling from field to field, waiting for crops to dry and equipment repairs (Supplementary Table S1).

### Crops and job tasks

Most of our study participants worked on nursery and landscape plantings (50.4%), followed by the melons crop category including watermelons, (28.1%; Supplementary Table S2). Other crops included tomatoes (22.2%), berries (11.9%), cucumbers (4.4%), and squash (1.4%). Thirty-two percent reported working on multiple crops, mostly among those who reported working on berries (77%), tomatoes (77%) and melons (92%). The most common job tasks were loading (52.6%), planting (46.7%), weeding (45.9%) and harvesting (38.5%). Fifty-four percent reported two or more tasks. Fifteen percent reported other tasks including cleaning, packing, quality control, sorting, stacking and supervising.

### Housing characteristics

Study participants lived in a variety of housing types (Table 3). Sixty-seven percent lived in labor camps with either barracks style units or that had multiple buildings on one property that housed workers. Twenty-four percent lived in single-family houses, 4.4% lived in trailers/mobile homes and 3.7% lived in motels. On average, the number of workers sleeping at each housing location was 18, number of bedrooms was 8, number of bathrooms 3.8 and number of kitchens was 2.

**Table 3:** Housing characteristics (*n* = 135).

Housing characteristics	*n*	% or Mean (SD)
Housing type		
Single-family house	33	24.4%
Hotel/motel	5	3.7%
Trailer/mobile home	6	4.4%
Barracks/labor camp	91	67.4%
Housing owned by employer (Y)	129	96.3%
Choose to live at this location (Y)	53	39.3%
Pay for this housing (Y)	5	3.7%
Average rent per month $		$210
Number of bedrooms		7.8 (7.9)
Multiple beds (Y)	130	97.0%
Number of workers sleeping at location		17.9 (25.6)
Number of bathrooms		3.8 (5.4)
Number of kitchens at location		2.0 (4.3)
Employer provides meals (Y)	36	26.7%
Worker pays for meals (Y)	32	91.4%
Time living at this location		
0 to less than 4 weeks	38	28.2%
4 to less than 8 weeks	45	33.3%
8 to less than 12 weeks	14	10.4%
12 to less than 16 weeks	15	11.1%
16 or more weeks	18	13.3%
Decline	5	3.7%

Ninety-six percent of housing was owned, controlled and provided by the employer. Fifty percent of participants reported that they had no choice in their housing location. Only 3.7% reported that they paid for housing and this was mostly among those living in trailers/mobile homes with 4–5 persons per trailer. The average monthly rent for the trailers was $210 per person.

More than 95% of participants had the following basic amenities in their housing (Supplementary Table S3): hot and cold water, toilets, shower/tub and refrigerators. Cooking facilities were either a stove (25%) and/or a hot-plate (68%). Fewer reported having central air conditioning (30%), AC window units (56%), fans (56%), washing machines (67%) and dryers (51%). Participants reported that AC window units or fans were not provided by the employer- some workers bring their own. Only 17% reported that they had a landline telephone or cellular phone at their housing.

In addition to documenting the presence or absence of basic amenities, our field notes and photos captured varying quality of amenities, furnishings and building structures. Shower stalls in multiple housing locations had broken concrete and some were covered with mold. At a large labor camp, there were posted signs in the large barrack style bathrooms warning residents not to drink the water. Residents reported to our team that these cement block bathroom interiors were unbearably hot. In another labor camp we observed broken and non-functional kitchen cabinetry, overflowing metal garbage cans and grounds littered with trash, make-shift electrical wiring, outdoor washing machines, and broken window screens which were useless against the relentless flies.

### Transportation

The vast majority of our study participants (>80%) reported relying on their employer not only for transport to the worksite, but also for activities of daily living such as shopping for food (79%) and accessing medical care (76%; Supplementary Table S4). Only four participants reported accessing health care via mobile clinics that visit housing sites.

### Pesticide exposures and hygiene practices

Workplace hazards such as pesticides can exacerbate respiratory illness. While few (7%) reported directly mixing, loading and applying pesticides, 37% of participants reported breathing pesticides in the air, 43% reported touching plantings with pesticide residues and 12% reported being sprayed with pesticides by a plane or tractor (Supplementary Table S5). Eight percent reported they had entered fields within 4 h of pesticide treatment. Nearly all participants (95%) reported having access to hand washing facilities at their worksites and toilets within 1/4 mile walking distance in the field, but only 67% reported access to drinking water. Sixty-seven percent reported receiving pesticide safety training at their current worksite – most commonly by video (79%). When asked in which language the training was provided, of those that responded, 92% stated Spanish, 4% stated Haitian Creole and 2% stated English. Despite receiving training, only 63% reported removing their work boots before entering their housing and 78% washed work clothes separate from other clothes.

### Heath behaviors and health conditions

As shown in Table 4, participants reported an average of 7 h of sleep during the week, compared to 8 h during the weekend. Nearly 18% reported ever smoking cigarettes (an estimate of lifetime exposure). Among those reporting smoking, the average number of years smoking was 8 years. When answering the survey questions about smoking, one participant commented that they do not really smoke when in Mexico, but tend to smoke more here in the United States to deal with stress that happens (only) here. On average, participants reported drinking alcoholic beverages 2 days per week and five drinks per day in the past week. Nearly 9% of study participants reported having morning cough and nearly 8% reported morning phlegm. The most reported chronic health conditions by participants were hypertension (20%) and diabetes (8%). Thirty-six participants (28%) reported taking prescription medications. Of those, the most common medications named were for controlling hypertension (47%), diabetes (15%) and analgesics (9%). Other medications included gastrointestinal medication (4%), epilepsy medication (2%) and anti-biotics (2%).

**Table 4:** Health behaviors and health conditions.

Health behaviors and conditions	n*	% or Mean (SD)*
Behaviors		
Smoke cigarettes		
Yes	24	17.8%
No	102	75.6%
Decline	6	4.4%
Years smoked	19	8.0 (6.0)
Number of cigarettes/day	19	10.0 (11.0)
Number of days per week drink alcoholic beverage	112	2.0 (7.0)
Number of alcoholic drinks per day	45	5.0 (5.0)
Sleep hours per day (week)	128	7.0 (2.0)
Sleep hours per day (weekend)	125	8.0 (3.0)
Taking any prescription medication (yes)	36	28.6%
Chronic health conditions		
Morning cough	11	8.5%
Morning phlegm	10	7.6%
Wheezing or whistling in your chest in the past 12 months	8	5.9%
Chest tightness in the past 12 months	7	5.1%
Cough at night	12	8.9%
Shortness of breath	3	2.2%
Asthma	0	0%
COPD	0	0%
Allergic rhinitis	2	1.6%
Chronic bronchitis	0	0%
Emphysema	0	0%
Other lung disease	1	0.8%
Diabetes mellitus	10	7.9%
Hypertension	24	19%
Stroke	0	0%
Coronary heart disease	0	0%
Chronic kidney disease	1	0.8%
Other heart disease	2	1.6%
Chronic liver disease	0	0%

* % based on the number of responses for each variable.

### Associations

In Tables 5 and 6, we show the distribution of dichotomized wages (<$15 vs. ≥ $15/h) and housing types by gender, country of origin, education, visa status, years of seasonal farm work, and each work task. All demographic characteristics and most work tasks were statistically significantly related to hourly wage rate. Specifically, 84% of male farmworkers received wages ≥$15/h. compared to 31% of female farmworkers (*p* < 0.001). A higher percentage of farmworkers on H-2A visas (93%) were paid $15/h. or more compared to U. S. citizens (75%), U.S. permanent residents (17%) and TPS beneficiaries (25%) (*p* < 0.001). H-2A farmworkers in comparison to all others (non-H-2A farmworkers) were many times more likely to report hourly pay of $15/h. or more (unadjusted OR 87, 95%CI: 15.54,829.26). Additional simplified comparisons using dichotomized wage outcomes however did not yield consistent patterns across work authorization groups. Using a Bonferroni correction for 6 pairwise comparisons (α ≈ 0.0083), we found individual comparisons H-2A vs. TPS (*p* < 0.001) and H-2A vs. permanent resident (*p* < 0.001) remained statistically significant. The H-2A vs. U.S. Citizen comparison was inconclusive (*p* = 0.11), likely due to small sample size.

**Table 5:** Association of wages with participant demographics, work authorizations and work tasks (*n* = 135).

	Wages		Fisher’s exact test *p*-value
	% <$15/h	% ≥$15/h	
Gender			<0.001
Male	9.02%	84.4%	
Female	53.9%	30.8%	
Country of origin			<0.001
Mexico	5.5%	89.1%	
Haiti	52.4%	28.6%	
United States (incl Puerto Rico)	0%	100%	
Other*	100%	0%	
Education			0.025
Less than primary school (grades 0–5)	12.5%	87.5%	
Primary school (grade 6)	28.0%	68.0%	
Secondary school (grade 9)	9.1%	87.9%	
Preparatory school (grade 12) or greater	11.8%	70.6%	
Declined	0%	1.0%	
Work authorization status			<0.001
H-2A visa	2.0%	93.3%	
TPS EAD	55.0%	25.0%	
U.S. citizen	25.0%	75.0%	
Permanent resident	67.0%	16.7%	
Number of years doing seasonal farmwork			<0.001
Less than 2 years	13.3%	66.7%	
2–3 years	18.5%	70.4%	
4–7 years	32.0%	64.0%	
8 or more years	3.1%	95.3%	
Decline	33.3%	0%	
Work tasks+			
Harvesting/picking	4.0%	96.0%	0.008
Pulling weeds	5.0%	95.0%	0.005
Loading	10.0%	90%	0.199
Planting	4.9%	95.1%	0.004
Driving vehicle	5.4%	94.6%	0.092
Setting up stakes	7.1%	92.9%	0.359
Driving a tractor	5.1%	94.9%	0.056
Irrigating	2.9%	97.1%	0.024
Apply pesticides	0%	100%	0.592
Pruning	4.8%	95.2%	0.032
Other	45.0%	40.0%	<0.001

+Fisher’s exact test was used to assess the difference in distribution of wage categories between those who reported each task and those who did not.

**Table 6:** Associations of housing characteristics with participant demographics, work authorizations and work tasks (*n* = 135).

	Single-family house	Hotel/motel	Trailer/mobile home	Barracks/labor camp	Fisher’s exact test *p*-value
Gender					<0.001
Male	26.0%	2.0%	0%	71.0%	
Female	8.0%	15.0%	46.0%	31.0%	
Country of origin					0.002
Mexico	30.0%	3.0%	4.0%	63.0%	
Haiti	0%	5.0%	0%	95.0%	
U. S. (incl Puerto Rico)	0%	0%	33.0%	67.0%	
Other	0%	0%	100.0%	0%	
Education					0.089
Less than primary school (grades 0–5)	0%	0%	0%	100.0%	
Primary school (grade 6)	8.0%	0%	0%	88,0%	
Secondary school (grade 9)	26.0%	3.0%	5.0%	67.0%	
Preparatory school (grade 12) or greater	38.0%	6.0%	6.0%	47.0%	
Declined	50.0%	0%	0%	50.0%	
Work authorization status					<0.001
H-2A visa	31.0%	3.0%	0%	66.0%	
TPS EAD	0%	5.0%	0%	95.0%	
U.S. citizen	25.0%	0%	25.0%	50.0%	
Permanent resident	0%	0%	83.0%	17.0%	
Number of years doing seasonal farmwork					<0.001
Less than 2 years	47.0%	0%	0%	53.0%	
2–3 years	59.0%	4.0%	11.0%	26.0%	
4–7 years	12.0%	12.0%	0%	76.0%	
8 or more years	8.0%	0%	5.0%	86.0%	
Decline	0%	0%	0%	1.0%	
Work tasks+					
Harvesting/picking	10.0%	2.0%	0%	87.0%	<0.001
Pulling weeds	42.0%	5.0%	0%	52.0%	<0.001
Loading	35.0%	4.0%	0%	59.0%	<0.001
Planting	40.0%	5.0%	0%	54.0%	<0.001
Driving vehicle	43.0%	0%	0%	57.0%	0.014
Setting up stakes	39.0%	0%	0%	61.0%	0.221
Driving a tractor	61.0%	5.0%	0%	32.0%	<0.001
Irrigating	31.0%	0%	0%	69.0%	0.391
Apply pesticides	50.0%	0%	0%	50.0%	0.519
Pruning	53.0%	2.0%	0%	44.0%	<0.001
Other	10.0%	0%	25.0%	65.0%	<0.001

+Fisher’s exact test was used to assess the difference in distribution of housing categories between those who reported each task and those who did not.

Housing type was statistically significantly associated with gender, country of origin, work authorization/visa status, years of seasonal farm work and most work tasks. Of note, a higher percentage of workers with work authorization as TPS beneficiaries (95%) were residing in barrack/labor camps. A higher percentage of female farmworkers (46%) were living in trailers/mobile homes compared to male workers (0%).

## Discussion

In this article, we provided an overview of the RESPIRAR Project, a study designed to better understand the social and structural and institutional drivers of respiratory health among MSFWs. Additionally, we presented baseline demographics of migrant and seasonal farmworkers living and working on Maryland’s Eastern Shore who were participants in the RESPIRAR Project longitudinal study 2023–2024. We delineated housing, work and wage characteristics, usual modes of transportation, health behaviors and physical health status. Lastly, we examined associations between demographics, wages and housing characteristics. Our objective in reporting on baseline data from the longitudinal study is to provide contemporary data on MSFWs in Maryland, since this information is lacking. To our knowledge, our study is the first to quantitatively report on Maryland MSFW demographic characteristics and housing since an enumeration study of Maryland MSFWs conducted in 2000 (7) and a survey of migrant farmworker housing in the eastern United States in 1997–1998 (81). Our baseline survey results provide insights our team will examine further using deeper community engagement through CBSD workshops with farmworkers and other stakeholders across the Mid-Atlantic. Further, the results have provided context and substantive grounding for ongoing legal analysis and policy surveillance of relevant state laws that influence respiratory health protections for farmworkers. While our empirical focus is Maryland and the Mid-Atlantic region in the U.S., our application of system dynamics and group model building to explicate the specific structural mechanisms contributing to farmworker ill health could be adapted by researchers outside the U.S. given the similarities in the exploitation and housing conditions among agricultural guest workers in other countries (17, 82–86).

We found that MSFWs on Maryland’s Eastern Shore hold a variety of work authorization statuses (e.g., H-2A and EAD under TPS), report Spanish and Haitian Creole as primary languages, have long tenure in agricultural work, often for the same employer. We also found disparities in wages by gender, education, and nationality (e.g., male, H-2A and Mexican workers were significantly more likely to earn ≥$15/h.). The majority of our MSFW participants were hired to work on nursery and landscape plantings and watermelon crops. Participants lived in various overcrowded housing types including detached/single family homes, motels, barrack/labor camps and mobile homes in very remote locations often on the property of farm owners/employers. Some housing locations lacked amenities such as air conditioning and laundry facilities. Visual inspections of these housing locations showed unsurprisingly bad conditions in some cases. Participants reported high rates of reliance on their employers for transportation for practically all needs of daily living - an example of built-in structural dependence and isolation. A substantial proportion of participants reported smelling pesticides in the air or touching plantings with pesticide residues while a low percentage of workers reported adhering to proven pesticide hygiene practices (e.g., removing boots before entering housing and washing work clothes separately). These findings are relevant to the overall goals of RESPIRAR given that pesticide exposures can exacerbate respiratory illness. Hypertension was the most common chronic health condition reported by participants but not everyone had access to needed medications. Chronic conditions such as hypertension and diabetes are known to increase the risks of lower respiratory infections including pneumonia, COVID-19, and influenza and contribute to more severe complications and hospitalizations. Fewer than 10% of participants reported chronic respiratory symptoms at baseline. Unsurprisingly, workers reported that they do not get paid when they cannot work due to illness or injury, incentivizing workers to continue working even while sick or injured. Farmworkers are generally excluded from Maryland’s mandatory paid sick leave laws (87, 88).

The characteristics of MSFW participants in our study are consistent with previous reports on the diversity of farmworkers in the Eastern Migratory Stream (22, 45) and other surveys on wages and housing conditions of farmworkers (45, 63, 89, 90), but also raise new questions from a structural perspective. The H-2A program is meant to help employers meet temporary labor needs for short/finite period of time such as during peak harvest time (91). However, in practice some employers rely almost entirely on H-2A workers (92, 93). Many of our H-2A study participants have been returning to Maryland for years often for the same employer, especially among the nursery workers who remain in Maryland for up to 11 months. This raises questions about the motivations and structural factors that drive the use of the H-2A program despite the reported expense and time incurred by employers to obtain the certification and hire workers. Notwithstanding the cost concerns, use of the H-2A program by agricultural employers has rapidly expanded in the last decade (6, 94), including in Maryland (5). With the recent federal changes (95) to the H-2A program to make it cheaper for employers to hire H-2A workers by allowing employers to set hourly deductions in worker wages for the provision of housing, it is likely that Maryland growers will employ more H-2A workers. Reports from farmworker advocates are already documenting the reduction in H-2A wages due to this policy change (96).

The presence of Haitian Creole speakers among migratory farmworkers is a characteristic that is unique to the Eastern Stream (22, 97) but is often overlooked by migrant health care organizations and governmental agencies responsible for enforcement of relevant policies such as the Migrant and Seasonal Agricultural Worker Protection Act (MSPA) in Maryland (e.g., lack of Haitian Creole translators; assuming Haitians do not did not fit the category of “migrant” because they were Black) (98). In our study, 15 % of our participants were from Haiti who mostly arrived in Maryland from Florida. Further, much of the existing public health research has mainly focused on the experiences of Hispanic or H-2A farmworkers (18, 29, 44). Thus, there is very little contemporary research on Haitian farmworkers in general and more specifically on Haitian migrant farmworkers employed in fruit and vegetable operations in Maryland (22, 98, 99). This gap is significant given that Haitian farmworkers occupy a structurally distinct position with the agricultural labor hierarchy. Ethnographic accounts from Maryland’s Eastern Shore document how Haitian workers, regardless of legal status, face differential treatment rooted in anti-Black racism rather than documentation alone, including fewer interpreters, less culturally responsive care, and exclusion from protections nominally available to other farmworker populations (98). This pattern is consistent with the broader history of the H-2 guestworker system, in which the displacement of Black domestic and Caribbean labor by Latino workers has been a defining, if underexamined, structural feature since the program’s origins (100), a dynamic that, as our own historical analysis of Maryland’s agricultural labor system shows, was not incidental but deliberately constructed (3, 101). In our sample, the wage disparity between Haitian TPS beneficiaries and H-2A workers reflects this broader structural pattern: TPS status confers work authorization but not the contractual wage protections attached to H-2A visas, leaving Haitian workers in a more precarious economic position despite comparable labor.

Despite statements by the agricultural industry that farmworkers are overpaid, analyses continually show that farmworkers in the United States earn relatively low wages (102). The Economic Policy Institute found that farmworkers earned 40% less than comparable nonagricultural workers in 2022 (103). Further, prior studies on wage inequalities among farmworkers have found that gender is a strong predictor of farmworker earnings with women earning consistently less than men (102, 104). This gender discrepancy has been attributed in part to women having less U.S. farm work experience, working fewer hours, more often being paid hourly than piece-wise, being less likely to work with pesticides, and being more likely to work on post-harvest tasks in comparison to their male counterparts (104). However these prior studies relied on data from the U.S. Department of Labor’s National Agricultural Worker Survey (NAWS), which by design does not include H-2A workers. In our study, we found not only gender wage disparities but also disparities by country of origin, education and visa status. In particular, we found that H-2A visa status was strongly associated with higher pay in comparison with U.S. permanent residents and TPS holders. We were not able to estimate multivariable models of wages that simultaneously controlled for work tasks due to sparse data across multiple task categories and resulting model instability. To address this, wages were dichotomized (<$15 vs. ≥ $15/h.), and we presented bivariate and task-adjusted models limited to a reduced set of predictors. H-2A workers must be paid at least the highest of the following rates: “the state’s ‘adverse effect wage rate’ or AEWR (set by the U.S. Department of Labor); prevailing wage for the region; Federal minimum wage; applicable state or local minimum wage ($15/h. in Maryland); or applicable collective bargaining wage (105). The AEWR has been historically higher than both federal and state minimum wages in most states. This stems from its original purpose to protect the wages of domestic U.S. farmworkers from being “adversely affected” by the employment of H-2A workers, given that U.S. workers in corresponding employment (working alongside H-2A workers) must be paid at least the equivalent paid to H-2A workers. Our study adds to farmworker literature on wages by including H-2A workers and those with work authorization as TPS beneficiaries. Our findings, particularly discrepancies between wages paid to H-2A and other workers, underscore the need to address the wage gap among farmworkers but also raises questions of whether the AEWR is truly protecting U.S.-based workers or is actually suppressing wages for all hired agricultural labor. More research is needed in this regard.

Prior studies of MSFW housing in the United States, including H-2A housing, report crowded or extremely crowded conditions (22, 42, 71, 106–108), structural and other deficiencies (71, 81, 109), housing regulation violations (63, 71, 110), pest infestation (63, 111, 112), and exposure to agricultural pesticides (113, 114). Numerous reports have documented how substandard housing creates severe health risks for farmworkers, from respiratory diseases and gastrointestinal problems to heat-related illnesses and heightened vulnerability to infectious diseases (18–20, 25, 98, 101, 115–118). We found that Eastern Shore MSFW housing types and conditions varied. Housing types included detached “single-family homes,” trailers/mobile homes, barracks/labor camps and motels. On average, 2–3 workers shared a bedroom across all housing locations in our study, exceeding the U. S. Department of Housing and Urban Development’s definition of severe overcrowding of 1.5 persons per room (excluding kitchens and bathrooms) (119). Overcrowding increases health and safety concerns (risk of spreading respiratory diseases, poor ventilation, creating a moist environment that encourages mold and pests infestation, etc.) and stresses the condition of the housing stock and infrastructure. However these overcrowded conditions are acceptable under the OSHA regulations for migrant labor housing. At one housing location in our study, 10 men slept in bunkbeds in one 23 ft. x23ft room which barely met the OSHA standard of 50 square feet per person under migrant labor housing regulations (120). From a respiratory heath perspective, specifying minimum requirements for just the floor space is not sufficient. Appropriate level of ventilation or air exchange (e.g., 15–20 cubic feet per minute of outdoor or filtered air per person) should be required (121). We also noted gender disparities in type of housing. Women workers were more likely to live in trailers in more crowded conditions with 4 persons per trailer. Overall living conditions among our study participants were generally poor with run down and broken furnishings, extensive mold in showers, structural damage and persistent fly and rodent infestation. Prior studies have found that substandard housing is more common among migrant farmworkers in the eastern U.S. than farmworker dwellings in the U.S. as a whole (108).

In addition to physical conditions of MSFW housing, the spatial isolation of the housing also functions as a structural determinant of health for farmworkers. In our study nearly 80% of participants reported relying on their employers for transport to the worksite, for shopping and accessing medical care. This reliance highlights the spatial isolation often experienced by MSFWS and implications for health (3, 69, 98, 122, 123). Several miles distances from the nearest medical facility and lack of access to public transit or other independent means of transportation increase the risk of worsening outcomes if a farmworker becomes ill or injured (98). Limited mobility also restricts farmworkers’ opportunities to forge social connections with local communities. Isolation of MSFW housing has been associated with mental health outcomes (122, 123). But this isolation is not by accident and it is actively maintained by employers by restricting outside access. During our field study we observed “No Trespassing” signs at some housing locations and observed a motel owner restricting movement of MSFWs housed there through his verbal threats and refusing visitors from social service organizations. At the same time farm employers assert they have right to approach workers at their housing sites at any time. Spatial arrangements of MSFW housing serve both economic exploitation and confinement as highlighted by scholarship on racial capitalism and critical geography which explicates how racial hierarchies are maintained through geographic segregation and containment (124, 125). Thus simply strengthening OSHA and ETA minimum standards for MSFW housing or increasing housing inspections may not fully disrupt these spatial dynamics that enable employers to exert control and undermine the human rights and dignity of farmworkers.

### Implications for next phase of RESPIRAR

The overall goal of the RESPIRAR Project is to illuminate how increased health harms, specifically infectious respiratory illnesses, among farmworkers are situated within broader systems of labor, immigration, agriculture and public health, and how these systems interact in order to inform actionable policy recommendations. The next phase our research involves applying system dynamics and group model building, novel applications of systems science methods which are is increasingly being recognized for their utility as “structural approaches” in public health research and practice and in pushing analyses of health inequities further “upstream” (126–128). System dynamics (SD), a systems science methodology, uses informal maps and formal models with computer simulation to elucidate how counterintuitive behaviors of systems and unanticipated consequences of policy and program interventions, such as disparate population health outcomes and persistent community health harms, may be explained by system structure, including resource accumulation and information delays, and formal and informal institutional rules and practices (129). A hallmark of the SD approach is the integration of multiple sources of structural data to represent complex systems through the use of causal loop diagrams (CLDs) and other qualitative mapping approaches. Community-based system dynamics (CBSD) is a tradition of SD that is grounded in participatory appraisal techniques that provides a framework and methods to explicitly engage those most impacted by policy decisions as co-equal partners in the system mapping and modeling processed (55). The CBSD approach focuses on centering the voices and priorities of communities, while investing in developing capacities of community members in the use of SD tools (55).

Our baseline survey results provide grounding into the current lived experiences of MSFWs in Maryland and aspects of multiple systems that we will interrogate further through CBSD group model building workshops co-facilitated with CATA, and ethnographic interviews (68, 130, 131). Specific findings from the baseline survey are informing the configuration and framing for upcoming workshops with farmworkers and other stakeholders. The disparities we observed by visa/work authorization status and gender are structured by labor and immigration policies at the Federal level and suggest a priority of engaging male and female farmworkers, and H-2A and non-H-2A workers, separately in our CBSD workshops in order to illuminate specific experiences and dynamics of housing conditions, wages, advocacy, and help seeking. Further, these disparities suggest a need to explore decision processes through which employers/growers prioritize sources of labor, request H-2A workers and set wages. Observations of current MSFW housing conditions suggest gaps between minimum safety standards (OSHA and ETA standards for migrant labor housing) as required under federal laws (Migrant and Seasonal Agricultural Worker Protection Act (MSPA) and the Immigration and Nationality Act (INA)) and actual implementation and enforcement of those regulations through state and local departments of health and labor as well as underlying motivations and norms that maintain spatial isolation of MSFWs, all critical topics for our planned CBSD workshops. Additionally, baseline survey results on pesticide safety practices highlight the underappreciated interconnections across policy domains and how deficiencies (e.g., lack of laundry facilities at employer-provided housing) in one domain (e.g., OSHA and ETA migrant labor housing standards) undermine the intended outcomes in another domain (e.g., EPA’s Worker Protection Standard on pesticide exposures). Workshops will separately engage farmworkers, as well as representatives of state and local agencies, to understand the mechanisms of implementation and enforcement, as well as sources of friction and resistance, operating across multiple regulatory and policy domains, that impede widespread achievement of improved living and working conditions for farmworkers. Therefore, our planned CBSD workshops with farmworkers, service providers, farmworker advocates, policy makers and regulators and interviews with growers provide a unique opportunity to build on empirical findings from this stage of research to disentangle the multiple pathways through which immigration, labor and occupational health and safety policy and regulatory actions influence and interact with goals, norms, priorities, and strategies of the communities and organizations that are subject to those regulations. By illuminating these complex social causal structures and pathways, we will develop more nuanced and contextually grounded understanding of how public policies, practices and norms may lead to avoidance or unintended consequences that hinder effective implementation, and identify system-level opportunities for improving the health and wellbeing of MSFWs.

### Strengths and limitations

Results of our baseline survey should be interpreted with a few limitations in mind. First, all of our study participants had self-reported work authorization. Therefore the demographic trends presented should not be applied to farmworkers without authorization. Second, migrant and seasonal labor on Maryland’s Eastern Shore include seafood and chicken processing workers who also suffer poor work and housing conditions (70, 132). However under the U.S. Department of Labor definitions (133), these workers are not considered agricultural workers, thus regulations on MSWF housing either under MSPA or the INI do not apply to these workers. With this distinction in mind, we designed our study to specifically focus on MSFWs who fit the U.S. Department of Labor definition of migratory agricultural work and/or were employed as H-2A workers so that we could directly link living and working conditions to structures created by the law. Further, caution must be taken when interpreting the results on “usual number of days worked per week”, “hours worked in the last week” and specific tasks as reported by participants since these results may depend on the timing of the baseline survey. We report the timing of the completed surveys - early season, mid-season and late season - so readers can make appropriate interpretations. Lastly, we acknowledge low participation rate by housing location and that our study sample was a convenience sample. We may have missed farmworkers whose living conditions may be more or less similar to our participants. However, the demographics of our participants in terms of country of origin, gender and visa status appear to be in line with current analyses of Eastern Migratory Stream farmworkers. Therefore, despite these limitations, we have confidence that our results are representative of Maryland’s Eastern Shore MSFWs who have work authorization.

Our study has a number of strengths. Most public health research on farmworkers has generally taken place in western and southern states, such as Florida, Texas, North Carolina, and California - we have referenced this prior work. Thus, we know little about key issues affecting farmworkers in other states, particularly in the Mid-Atlantic States. Our study fills that gap by providing baseline demographics of migrant and seasonal farmworkers living and working on Maryland’s Eastern Shore. The community-based participatory research approach is also a strength. The partnership with CATA ensures that study design and results are beneficial to the needs of the farmworker community served by CATA and supports trust building with farmworkers. Data from baseline survey have been instrumental in communicating concerns about farmworker housing conditions to other farmworker advocacy groups, state law makers and regulatory agencies in Maryland. Our results are timely given the recent changes by the U.S. Department of Labor to H-2A program rule allowing employers to deduct a set hourly amount from H-2A worker hourly wages for the provision of housing (95). We will be able to interrogate the impact of these structural changes through our engagement with farmworkers and other stakeholders in our CBSD workshops. Our systems and legal analysis will benefit from evidence based on the lived-experience of our study participants.

## Conclusion

Impoverished working and living conditions of MSFWs are constant features of the U.S. agricultural labor system (3, 101). Public health research tends to focus on these conditions and harms at the individual level rather than on the systems that create and sustain these harms. We contend that these conditions are deliberately produced features of the migrant and seasonal agricultural labor system itself. The goal of the RESPIRAR Project is to apply a systems approach to examine how the structures of law, policy, practices and norms interact to give rise to the lived experiences we have documented through our field study and the baseline survey. Results from our study will inform the design of policies and best practices to achieve better alignment between public health, social services and labor systems to optimize living and working conditions for better health protections among these invisible and vulnerable workers.

## Data Availability

The data are not publicly available due to their containing information that could compromise the privacy and safety of research participants. Requests to access the datasets should be directed to devonps@umich.edu.
