# Supplementary material for: Fields of inequity: initial insights into structural disparities among migrant farmworkers in Maryland
Source: Front Public Health. 2026 Oct 1;14:1930068. doi: 10.3389/fpubh.2026.1930068 (PMC13668501; doi:10.3389/fpubh.2026.1930068)
Supplement: Supplementary file 1 [file Table_1.DOCX]

**Table S1.  Situations when not able to work and if paid (n=135)**

|  | Not able to work  n (%)  *report the Yes responses here* | Not Paid*  n (%)  *report the No responses here* |
| --- | --- | --- |
| Traveling from field to field | 63 (47.0%) | 1 (1.6%) |
| Waiting for crops to dry | 38 (28.4%) | 8 (24.0%) |
| Waiting for equipment to be repaired | 59 (44.0%) | 5 (11.0%) |
| illness or injury | 29 (21.6%) | 18 (62.0%) |
| When it is too hot | 38 (28.6%) | 13 (34.0%) |
| Too much rain or flooding | 38 (28.2%) | 26 (68.0%) |
| Thunderstorm or lightning | 27(29.3%) | 23 (85.0%) |
| Air quality is bad | 9(9.8%) | 3 (33.3%) |

*based on number of responses
