# Supplementary material for: Fields of inequity: initial insights into structural disparities among migrant farmworkers in Maryland
Source: Front Public Health. 2026 Oct 1;14:1930068. doi: 10.3389/fpubh.2026.1930068 (PMC13668501; doi:10.3389/fpubh.2026.1930068)
Supplement: Supplementary file 2 [file Table_2.DOCX]

**Table S2. Crop Types and Tasks** (n=135)

| Crops | |
| --- | --- |
|  | n (%)* |
| Berries | 16 (11.9%) |
| Tomatoes | 30 (22.2%) |
| Green Peppers | 1(0.7%) |
| Squash | 2 (1.4%) |
| Cucumbers | 6 (4.4%) |
| Melons incl watermelons | 38 (28.1%) |
| Nursery and  Landscape Plantings (e.g. trees/seedlings, shrubs, flowers) | 68 (50.4%) |
| Other** | 7 (5.2%) |
| Multiple Crops*** | 32 (23.7%) |
| Decline | 3 (2.2%) |

| Tasks | |
| --- | --- |
|  | n (%)* |
| Harvesting/picking | 52 (38.5%) |
| Pulling weeds | 62 (45.9%) |
| Loading | 71 (52.6%) |
| Planting | 63 (46.7%) |
| Driving Vehicle | 37 (27.4%) |
| Setting up stakes | 28 (20.7%) |
| Driving a tractor | 41 (30.4%) |
| Irrigating | 35 (26%) |
| Apply pesticides | 6 (4.4%) |
| Pruning | 45 (33.%) |
| Other**** | 20 (14.8%) |
| Multiple Tasks | 73 (54%) |
| Decline | 2 (1.5%) |

*% based on number of responses

** Other crops included corn and pumpkins

***Working on multiple crops were reported mostly among those who reported working on berries (77%), tomatoes (77%) and melons (92%)

**** cleaning, packing, quality control, sorting, stacking and supervising.
