# Supplementary material for: Fields of inequity: initial insights into structural disparities among migrant farmworkers in Maryland
Source: Front Public Health. 2026 Oct 1;14:1930068. doi: 10.3389/fpubh.2026.1930068 (PMC13668501; doi:10.3389/fpubh.2026.1930068)
Supplement: Supplementary file 3 [file Table_3.DOCX]

**Table S3.  Housing Amenities (n=135)**

|  | Do you have it  n (%) Yes | Does it work  n (%*) Yes |
| --- | --- | --- |
| Hot Water | 129 (95.56%) | 124 (96.1% ) |
| Cold Water | 132 (97.8%) | 128 (100%) |
| Toilet | 132 (97.8%) | 126 (95.5%) |
| Shower/Tub | 131 (97.8%) | 124 (94.7%) |
| Stove | 99 (75%) | 95 (96%) |
| Hotplate | 92 (68.1%) | 90 (97.8%) |
| Fridge | 131 (97.8%) | 123 (93.9%) |
| Central Heating | 69 (52.3%) | 63 (91.3%) |
| Central AC | 40 (29.9%) | 39 (97.5%) |
| AC Window Unit | 75 (56.4%) | 73 (97.3%) |
| Fans | 74 (56.1%) | 69 (93.2%) |
| Laundry Washer | 93 (68.9%) | 88 (94.6%) |
| Laundry Dryer | 67 (50.8%) | 63 (94%) |
| Telephone (landline or cellular) | 22 (16.5%) | 20 (90.9%) |

*% based on the number of responses for each amenity

Note: Some participants reporting AC units informed field teams that they bring their own AC window units. Cooling is not required in the Federal ETA or OSHA standards for migrant labor housing.
