# Supplementary material for: Fields of inequity: initial insights into structural disparities among migrant farmworkers in Maryland
Source: Front Public Health. 2026 Oct 1;14:1930068. doi: 10.3389/fpubh.2026.1930068 (PMC13668501; doi:10.3389/fpubh.2026.1930068)
Supplement: Supplementary file 4 [file Table_4.DOCX]

**Table S4.  Transportation (n=135)**

| Modes for Transportation | n (%) or Mean (SD) |
| --- | --- |
| For Work* |  |
| Ride/“raite” (carpool) | 7 (5.2%) |
| Own vehicle | 4 (3%) |
| Friend/relative | 3 (2.2%) |
| Boss, contractor or  farmer | 91 (67.4%) |
| Other rideshare | 0 (0%) |
| Bus or van of the ranch/farm | 34 (25.2%) |
| Public bus | 1 (0.7%) |
| Walk or go on bicycle | 1 (0.7%) |
| Ride carpool and Boss/contractor/farmer |  |
| Boss/contractor/farmer or  Bus/van/ranch | 125 (93%) |
| Time in transportation to work (min) | 13 min (9min) |
| Pay for transportation to Work (Y) | 1 (0.8%) |
| For Shopping* |  |
| I pay for a ride | 6 (4.4%) |
| Own vehicle or friend | 10 (7.4%) |
| Public Transit | 3 (2.2%) |
| Walk or bike | 0 (0%) |
| Company/employer | 105 (79.0%) |
| Other | 8 (7.0%) |
| For Medical Care* |  |
| I pay for a ride | 10 (7.4%) |
| Own vehicle or a friend/relative | 8 (5.9%) |
| Public transit | 2 (1.4%) |
| Walk or go by bicycle | 0(0%) |
| The boss takes me | 82 (61.0%) |
| My crew leader takes me | 18 (13.3%) |
| Health care clinic provides transportation | 2(1.5%) |
| Mobile clinics that visit the worksite or housing site | 4 (3.0%) |
| Other | 6 (4.4%) |

*multiple responses were allowed
