# Supplementary material for: Fields of inequity: initial insights into structural disparities among migrant farmworkers in Maryland
Source: Front Public Health. 2026 Oct 1;14:1930068. doi: 10.3389/fpubh.2026.1930068 (PMC13668501; doi:10.3389/fpubh.2026.1930068)
Supplement: Supplementary file 5 [file Table_5.docx]

**Table S5.  Indicators of Pesticide Exposures and Protective Measures (n= 135)**

| Variable | n | % or Mean (SD)* |
| --- | --- | --- |
|  |  |  |
| Currently working in areas treated with pesticides |  |  |
| Yes | 68 | 50.0% |
| No | 62 | 46.0% |
| Don’t Know | 4 | 3.0% |
| Received training at current work site | 80 | 68.4% |
| How received training** |  |  |
| In person | 32 | 40.0% |
| By video | 63 | 79.0% |
| Given booklet | 14 | 18.0% |
| Loaded, mixed or applied pesticides | 9 | 7.0% |
| Cleaned or repaired containers or equipment used for applying or storing pesticides | 6 | 6.5% |
| Breathed pesticides in the air | 50 | 37.3% |
| Touched plants with pesticide residue | 57 | 43.0% |
| Been sprayed with pesticides by a plane or tractor | 17 | 12.7% |
| Entered the field within four hours after pesticide treatment | 11 | 8.2% |
| Field Sanitation Facilities |  |  |
| Handwashing | 127 | 95.0% |
| Drinking water | 92 | 67.0% |
| Disposable cups provided | 57 | 64.0% |
| Bathrooms nearby, within 1/4 mile walking distance | 127 | 95.0% |
| Hygiene Practices |  |  |
| Remove boots when entering home | 84 | 63.0% |
| Number of minutes after entering home from worksite change clothing | 100 | 12.7 (22) |
| Number of minutes after entering home from worksite take bath | 99 | 26 (23) |
| Washes work clothes separate from other clothes | 104 | 78.0% |

Note: *% based on the number of responses for each variable

** % based on the number of the participants who responded they received training. Participants could select all that apply
